# PACAP6-38 improves nitroglycerin-induced central sensitization by modulating synaptic plasticity at the trigeminal nucleus caudalis in a male rat model of chronic migraine

**DOI:** 10.1186/s10194-023-01603-3

**Published:** 2023-06-05

**Authors:** Lily Zhang, Yanjie Zhou, Liu Yang, Yue Wang, Zheman Xiao

**Affiliations:** 1 Department of Neurology, Renmin Hospital of Wuhan University, 99 Zhang Zhidong Road, Wuhan, 430060 Hubei Province China; 2 Central Laboratory, Renmin Hospital of Wuhan University, 9 Zhang Zhidong Road, Wuhan, 430060 Hubei Province China

**Keywords:** Migraine, Central sensitization, PACAP, PAC1R, Synaptic plasticity

## Abstract

**Aims:**

Chronic migraine (CM) is a common neurological disorder with complex pathogenesis. Evidence suggests that pituitary adenylate cyclase-activating peptide (PACAP) induces migraine-like attacks and may be potential a new target for migraine treatment, but the therapeutic results of targeting PACAP and its receptors are not uniform. Therefore, the aim of this study was to investigate the regulatory effect of PACAP type I receptor (PAC1R) antagonist, PACAP6-38, on nitroglycerin (NTG)-induced central sensitization in a CM model.

**Methods:**

Sprague–Dawley (SD) rats received repeated injections of NTG to construct a CM model. Mechanical and thermal thresholds were measured using Von Frey filaments and hot plate tests. C-Fos expression was measured by western blotting and immunofluorescence staining to assess the central sensitization. PACAP6-38 was intracerebrally injected into the trigeminal nucleus caudalis (TNC), and then the changes in c-Fos, the synaptic-associated proteins, phospho-ERK1/2 (p-ERK1/2), phosphorylation of cyclic adenosine monophosphate response element-binding protein (p-CREB) and brain-derived neurotrophic factor (BDNF) were detected. Transmission electron microscopy (TEM) and Golgi-Cox staining were used to observe the ultrastructure of synapses and dendritic structures of TNC neurons.

**Results:**

The results showed that PACAP and PAC1R expression were significantly raised in the TNC after repeated NTG injections. Additionally, PACAP6-38 treatment alleviated nociceptive sensitization, inhibited NTG-induced overexpression of c-Fos and synaptic-associated proteins in the TNC of CM rat, restored aberrant synaptic structures. Furthermore, the expression of ERK/CREB/BDNF pathway was depressed by PACAP6-38.

**Conclusions:**

Our results demonstrated that abnormal synaptic structure in the TNC of CM, which could be reversed by inhibition of PAC1R via down-regulating the ERK/CREB/BDNF signaling pathway. PACAP6-38 improves NTG-induced central sensitization by regulating synaptic plasticity in the TNC of CM rat, which may provide new insights into the treatments targeting PACAP/PAC1R in migraine.

## Introduction

Chronic migraine (CM) is a common debilitating neurological disorder manifested by headaches at least 15 days a month and migraines at least 8 days per month [1]. Based on epidemiological data, about 3% of episodic migraine (EM) patients transform to CM every year [2], resulting in a heavier personal and socioeconomic burden. However, there are limited treatment options available since its complex pathogenesis and poor treatment response [3].

The pathogenesis of migraine is not clear enough. Genetic susceptibility and activation of the trigeminal nervous system are thought to be the main pathophysiological factors of migraine. Central sensitization of trigeminovascular neurons in the trigeminal nucleus caudalis (TNC) contributes to the pathogenesis of hypersensitivity and chronicity in migraine [4]. Furthermore, the enhancement of membrane excitability and synaptic efficacy is essential for central sensitization in CM [5]. Activation of trigeminovascular pathway results in release of pro-inflammatory, vasodilatory, or pain producing neuropeptides, such as calcitonin gene-related peptide (CGRP), pituitary adenylate cyclase activating polypeptide (PACAP) [6]. Based on clinical and basic research, the neuropeptide CGRP has been considered to be central in migraine pathophysiology [7]. Furthermore, the success of clinical trials targeting CGRP and its receptors has raised the interest in other peptide-related drug targets for migraine therapy [8, 9].

PACAP is a member of vasoactive intestinal polypeptide (VIP)/secretin/glucagon peptide superfamily, and it exists in two forms: PACAP27 and PACAP38 (predominant form in the central nervous system (CNS)) [10]. Both isoforms are capable of binding to G-protein-coupled receptors (GPCRs) specific (PAC1) and less specific (VPAC1 and VPAC2) to PACAP [11]. Unless otherwise indicated, we will refer to PACAP38 as PACAP in this study. Patients with migraine without aura may experience migraine-like attacks following intravenous infusion of PACAP [12, 13]. Immunohistochemical evidence show that PACAP is primarily found in tissues associated with migraine pathophysiology such as trigeminal ganglia (TG) and TNC [14, 15]. PACAP co-localizes with CGRP in the trigeminovascular system [16], and both activate adenylate cyclase upon binding to receptors, with consequently increased formation of cyclic AMP (cAMP) [17]. A new study showed that knockout of CGRP in mice couldn’t prevent pain triggered by PACAP [18]. The findings indicate that the mechanisms by which PACAP induces migraine are independent of CGRP, meaning its potential to be an alternative therapeutic target for migraine patients who have a poor response to anti-CGRP therapy [18, 19].

There exist few predicative animal models of migraine due to its complex pathological mechanisms [20]. Since Pradhan et al. established a new model of CM with nitroglycerin (NTG) [21], NTG has been widely used to establish experimental models for animal studies of CM. NTG mediates persistent basal peri-orbital and hind-paw hypersensitivity which recover to baseline levels around 6–7 days after the last NTG administration [21]. PACAP is demonstrated to be an important mediator in NTG-induced trigeminovascular activation in mice [22]. The immunoreactivity of both PACAP and its receptors were upregulated in the TG and TNC of rats after NTG injection [23, 24]. Besides that, PACAP enhances hippocampal synaptic transmission and plasticity [25, 26], which can be blocked by PACAP6-38 [27]. In vitro studies showed that PACAP could increase the excitability of diverse neurons via activating extracellular signal-regulated kinase (ERK) [28, 29]. In addition, the major downstream mediators of ERK, cAMP response element-binding protein (CREB), has been regarded as an essential marker of central sensitization in CM rats [30, 31]. Phosphorylated CREB (p-CREB) can increase the expression of Brain-Derived Neurotrophic Factor (BDNF), enhancing synaptic transmission and long-term potentiation (LTP) [32, 33]. These data suggest that blocking PACAP/PAC1R may ameliorate NTG-induced abnormalities of synaptic transmission via the ERK/CREB/BDNF signaling pathway.

In this study, we explored the effects of the antagonist PACAP6-38 on NTG-induced central sensitization in a rat model of CM, primarily focusing on synaptic plasticity. Our data revealed that PACAP6-38 reduced neuronal activation in TNC and alleviate central sensitization in rat models of CM, and we suggest that these beneficial effects might be mediated by regulating synaptic plasticity involved ERK/CREB/BDNF signaling.

## Methods

### Animals

**Fig. 1:**
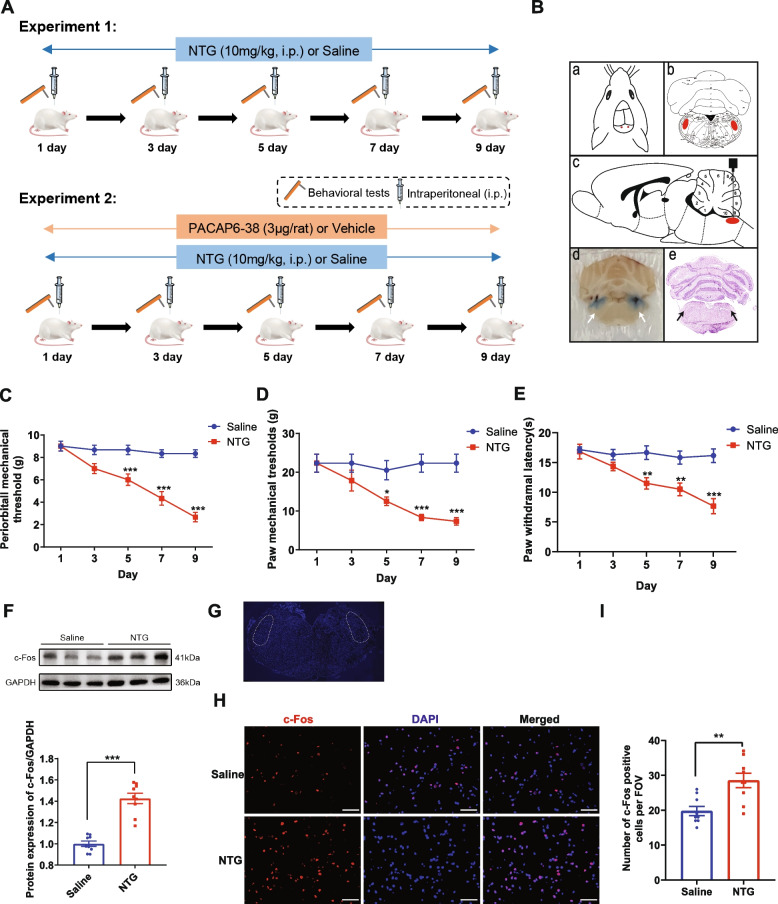
Repeated injection of NTG induced hyperalgesia and upregulated c-Fos expression. **A**. Experimental design and timeline. **B**. Schematic diagrams of injection sites. **a**-**c**. Injection sites and TNC location (red circles) are shown in coronal and sagittal sections according to the rat brain atlas adapted from Paxinos and Watson. **d**-**e**. Coronal sections of the rat brain with methylene blue injection and HE staining to verify the correct placement of guide cannulas (arrow). **C**-**E**. The basal mechanical pain threshold of periorbital (**C**) and hind paw (**D**) and the thermal pain threshold (**E**) of the hind paw in each group. Mean ± SEM, *n* = 6/group. Two-way ANOVA with the Bonferroni post hoc test, *p < 0.05, **p < 0.01, ***p < 0.001 compared with saline group. **F**. The protein expression of c-Fos was assessed by western blot and was markedly higher in the NTG group than in the saline group. **G**. The white dotted line frame indicates the TNC regions. Scale bar: 100 μm. **H**-**I**. Representative images (**H**) and quantitative analysis (**I**) of c-Fos-positive cells (red) in TNC of different groups. Immunofluorescence staining showed that the average numbers of c-Fos-positive cells in the NTG group were significantly increased compared to the saline group. Scale bar: 50 μm. FOV = 1.02 × 10^6^ µm^3^. Mean ± SEM, *n* = 3/group, two-tailed Student’s t-test, *p < 0.05, **p < 0.01, ***p < 0.001 compared with the saline group. Abbreviations: PACAP, pituitary adenylate cyclase-activating peptide; NTG, nitroglycerin; CM: chronic migraine

All animal procedures received approval from the Institutional Animal Care and Use Committee (IACUC) of Renmin Hospital of Wuhan University (No. 20201101). The work was also carried out in compliance with the ARRIVE guidelines. Male Sprague–Dawley (SD) rats (body weight 210–235 g; age 7 weeks) were obtained from the Laboratory Animal Centre of Renmin Hospital of Wuhan University and housed in the specific pathogen-free (SPF) conditions with a12-h light/dark cycle, humidity of 60 ± 5% and temperature of 22 ± 2 °C. Food and water were well provided. A total of 60 rats were used in this study. After a week of acclimation, the rats were randomly grouped using a random-number sequence and kept in polycarbonate cages (6 per cage). The experimental design schedule is depicted in Fig. 1A. In experiment 1, the rats were divided into two groups at random: (1) Saline and (2) NTG (*n* = 6/group). In experiment 2, the rats were assigned to four groups: (1) Saline + VEH, (2) Saline + PA6-38, (3) NTG + VEH, and (4) NTG + PA6-38 (*n* = 12/group). The investigators were blind to the grouping of animals.

### Surgical procedure

The surgical procedure was conducted as described previously [34, 35]. Rats were anesthetized with a ketamine-xylazine mixture (100 mg/kg ketamine, 10 mg/kg xylazine) and fixed on a stereotaxic apparatus (68,002, RWD Life Science). 26-gauge stainless steel guide cannulas (C315G, Plastics One) were implanted bilaterally in the TNC region (14.08 mm posterior to the bregma, 2.75 mm lateral to the midline, and 8.65 mm deep) base on the atlas of Paxinos and Watson [36]. The dummy cannula (C315DC, Plastic one) was inserted to prevent clogging. Subsequently, the cannulas were fixed with dental acrylic cement. At the end of the experiment, the correct position of the cannula was confirmed by injecting methylene blue (0.5 μl/side) through guide cannulas. Schematic diagrams of the stereotactic surgery and injection sites are shown in Fig. 1B.

### Microinjection

PACAP6-38 (HY-P0220A, MCE) was dissolved in 0.9% saline to make a working solution of 1 μg/μl. One week after recovery from surgery, rats received 3 μl of PACAP6-38 or vehicle (0.9% saline) every other day for 9 days through a 33-gauge internal cannula (C315I, Plastic one) extending 0.5 mm beyond the guide cannula. The injection cannula was connected to a 5-μl Hamilton syringe through PE-20 tubing with an injection rate of 1 μl/min. The dosage and approach of PACAP6-38 were based on previous studies [37, 38]. After each infusion, the cannula was kept in place for 1 min to spread the solution.

### Establishment of the CM model

After microinjection, the rats were left for 30 min to fully awaken from anesthesia. Subsequently, rats received intraperitoneal (i.p.) injection of nitroglycerin (NTG) to establish CM model according to Pradhan's study in 2014 [21]. Briefly, 5 mg/mL NTG (Runhong, Henan, China) stock solution was dissolved in 30% propylene glycol, 30% alcohol and water. The stock solution was diluted in 0.9% saline to a dose of 1 mg/ml before each injection. Since previous studies revealed that the vehicle (6% propylene glycol, 6% ethanol, 0.9% saline) did not produce any mechanical sensitivity with comparable effects to saline injection [21], rats received diluted NTG (10 mg/kg, i.p.) or an equivalent amount of saline every other day for total 5 times (Fig. 1A).

### Behavioral tests

Central sensitization is characterized by cutaneous allodynia concerning the craniofacial and noncraniofacial regions [20]. Thus, we assessed the condition of cutaneous hyperalgesia by measuring the mechanical threshold and plantar thermal sensitivity. All behavioral assessments were conducted between 10:00 am to 16:00 pm in a quiet environment. The rats were trained for 3 days prior to the experiment and acclimated for at least 30 min before the test. Behavioral tests were conducted by the investigators blinded to each group.

### Assessment of mechanical allodynia

As previously described [39, 40], mechanical allodynia was measured using calibrated Von Frey filaments (Aesthesio, Italy). For periorbital mechanical allodynia, Von Frey filaments were applied to the periorbital area perpendicular to the skin. If the rats quickly retracted their heads or scratched faces with front paws, it was considered a positive response. To test the paw withdrawal threshold, Von Frey filaments were pressed against the plantar surface of the hind paw. Flinching or shaking the paw was regarded as a positive response. If the rat exhibited a positive response, the filament intensity was reduced. Otherwise, the filament intensity was increased until the rat showed a positive response. The threshold was recorded as the minimum force to elicit a positive response and was averaged over three repeated tests. Each rat was assessed at least three times at a 2-min interval.

### Assessment of thermal hyperalgesia

For thermal nociceptive responses, the paw withdrawal latency in response to injurious thermal stimuli was recorded every other day before drug administration. The rats were kept on a flat plate inside a transparent hood for 30 min to acclimatize. Another identical hot plate was set at a temperature of 55 °C with a 30-s turn-off time to protect the rats. After placing the rat on the hotplate, the time of paw licking, lifting or jumping off the hotplate was recorded as paw withdrawal latency reflecting thermal hyperalgesia. The final thermal withdrawal latency was defined as the average of three recordings with a 5 min interval.

### Western blot analysis

The rats were euthanized with CO_2_ two hours after the administration of NTG on day 9, and the brain tissue was harvested. The TNC tissue was immediately separated on ice and stored at -80 °C for immunoblotting. The TNC tissue was lysed in RIPA (Servicebio, China) buffer containing PMSF (Servicebio, China) and a protease inhibitor cocktail (Servicebio, China). For measuring protein concentration, bicinchoninic acid (BCA) protein assay kits (Beyotime, China) were used, and samples were separated using PAGE gels fast preparation kits (Epizyme Biotech, China), then transferred to PVDF membranes (Millipore, USA). The PVDF membranes were blocked at room temperature with Protein Free Rapid Blocking Buffer (Epizyme Biotech, China) for 30 min and then incubated overnight with primary antibodies. The membranes were then washed with 1xTBST three times, and then incubated at room temperature for one hour with horseradish peroxidase (HRP)-conjugated secondary antibodies. Immunoreactive bands were detected using the Enhanced chemiluminescence substrate (ECL) substrate (Biosharp, China) and analyzed using Image J software. The Table 1 contains information on each antibody used for western blotting.

**Table 1:** Antibodies used in western blot and immunofluorescence staining assays

Antibody	Manufacturer	Number	Host	Dilution
**For western blot analysis**				
PACAP	Santa Cruz	sc-166180	Mouse	1:500
PAC1	Abclonal	A3120	Rabbit	1:1000
c-Fos	Proteintech	66,590–1-Ig	Mouse	1:2000
PSD95	Affinity Biosciences	AF7839	Rabbit	1:1000
SYP	Wanleibio	WL03058	Rabbit	1:1000
SYT1	Abclonal	A0992	Rabbit	1:1000
Phospho-ERK1/2 (Thr202/Tyr204)	Affinity Biosciences	AF1015	Rabbit	1:1000
ERK1/2	Affinity Biosciences	AF0155	Rabbit	1:1000
Phospho-CREB (Ser133)	Affinity Biosciences	AF3189	Rabbit	1:1000
CREB	Affinity Biosciences	AF6188	Rabbit	1:1000
BDNF	Wanleibio	WL0168	Rabbit	1:500
GAPDH	Proteintech	10,494–1-AP	Rabbit	1:3000
HRP conjugated anti-mouse	Servicebio	GB23301	Goat	1:3000
HRP conjugated anti-rabbit	Servicebio	GB23303	Goat	1:3000
**For immunofluorescence staining assays**				
PACAP	Santa Cruz	sc-166180	Mouse	1:50
PAC1	Abcam	ab28670	Rabbit	1:500
c-Fos	Servicebio	GB12069	Mouse	1:500
GFAP	Wanleibio	WL0836	Rabbit	1:100
NeuN	Proteintech	26,975–1-AP	Rabbit	1:300
Iba1	Wako	019–19,741	Rabbit	1:500
PSD95	Affinity Biosciences	AF7839	Rabbit	1:200
Dylight 594, Goat Anti-Rabbit IgG	Abbkine	A23420	Goat	1:200
Dylight 594, Goat Anti-Mouse IgG	Abbkine	A23410	Goat	1:200
DyLight 488, Goat Anti-Mouse IgG	Abbkine	A23210	Goat	1:200
Dylight 488, Goat Anti-Rabbit IgG	Abbkine	A23220	Goat	1:200

### Hematoxylin–eosin and immunofluorescence staining

The rats were perfused transcardially with 250 ml of 0.9% saline and 250 ml of 4% paraformaldehyde after deep anesthesia. Then, the brains collected were postfixed in 4% paraformaldehyde at 4℃ overnight and soaked in 20% and 30% sucrose solution for dehydration. TNC tissues were embedded in wax blocks and cut into 4 μm sections. The brain sections were stained with hematoxylin–eosin (HE) stain according to routine protocols. In addition, the sections were routinely dewaxed and hydrated before heat-induced antigen retrieval. The non-specific binding sites were blocked by 5% bovine serum albumin (BSA) solution for 1 h. After incubation with the primary antibodies at 4 °C overnight, the sections were incubated with secondary antibodies at room temperature for 1 h free of light. Finally, the nuclei were stained with DAPI (Biosharp, China). The images were visualized with a fluorescence microscope (Olympus BX53; Olympus, Japan). Image J was used to quantify the number of positive cells within a rectangular field of view (field of view, FOV, 240 × 180 µm^2^), which included three rats each group, with four to six FOVs per section. The Table 1 contains information on the primary and secondary antibodies used for immunofluorescence staining.

### Transmission electron microscopy (TEM)

After general anesthesia, the rats were perfused intracardially with 2.5% glutaraldehyde. TNC was rapidly collected and soaked in 4% glutaraldehyde at 4 °C overnight. Then, the TNC tissues were cut into 1mm^3^ segments, and sent to Servicebio Laboratory of Wuhan for post-fixing, embedding, sectioning, and staining. The images of synapses were taken by the Hitachi TEM system and analyzed with Image Pro Plus. The width of the synaptic cleft, the thickness of the postsynaptic density (PSD) and the curvature of the synaptic interface were measured according to the method previously reported [41, 42]. Five micrographs were arbitrarily taken from each rat using the Hitachi TEM (HT 7800 120kv) and the images were analyzed with Image Pro Plus. During the process of taking and analyzing the photographs, the investigators were blinded to the experimental groups.

### Golgi‑cox staining

The FD Rapid Golgi Staining kit (FD Neuro Technologies, Inc) was used for Golgi-Cox staining. The TNC tissues were collected and immersed in the staining solution for 14 days at room temperature with dark light (replace the new dyeing solution after soaking for 48 h and then replace the new staining solution every 3 days). The TNC tissues were cryostat-cut by vibratome to obtain 100 μm thick sections, and sections were pasted to gelatin-coated slides. The staining process was carried out according to the manufacturer’s instructions. The images were acquired using an electron microscope (Nikon E100, Japan), and the panoramic image of the TNC tissue was obtained by the 3Dhistech Pannoramic Scan. For dendritic spines analysis, pyramidal neurons in the TNC region fully penetrated by the Golgi coloration were selected. Finally, the blinded researchers counted the number of spines on at least 3 apical dendritic segments of 20 μm in length to measure spine density using Image-pro Plus software. Each group contained three rats and five images per rat were investigated.

### Statistical analysis

Statistical analysis and graph generation were performed using GraphPad Prism version 8.0. All data were analyzed as mean ± standard error of the mean (SEM), where *n* = number of animals. Group size for significance at α = 0.05 with 0.8 power was determined from previous studies and based on the 3Rs using G*Power analysis. Prior to statistical analysis, the Shapiro–Wilk (S-W) normality test and the Bartlett's test were applied to ensure normality and homogeneity of variance. Two-way ANOVA with Bonferroni post hoc test was used to analyze behavioral data. Two-tailed Student's t-test was used for statistical comparisons between two groups, and one-way ANOVA with Dunnett's test was applied for statistical comparisons among groups. *P* < 0.05 was considered statistically significant.

## Results

### Repeated NTG treatment induced hyperalgesia and upregulation of c-Fos in TNC

In this experiment, the CM model was established by repeated NTG administration (Fig. 1A). According to previous studies [21], this model produces central sensitization persisting for days after cessation of NTG administration. Compared with the rats treated with saline, the mechanical thresholds and the latencies to noxious heat declined gradually after NTG injection (Fig. 1C-E). On day 9, the mechanical and thermal thresholds in the NTG group remained significantly lower than those in the saline group (*p* < 0.001; Fig. 1C-E). C-Fos is thought to be a biomarker of neuronal activation responding to nociceptive stimuli, which is highly correlated with migraine pathogenesis [43]. Our results indicated that the protein expression of c-Fos in the TNC was upregulated significantly in the NTG group when compared with the saline group (*p* < 0.001; Fig. 1F). Compared with saline-treated rats, immunofluorescence staining revealed a marked increase in the density of c-Fos-positive cells in the TNC of NTG-treated rats (*p* < 0.01; Fig. 1G-I). These behavioral data and the upregulation of c-Fos were considered as indicated key indexes of central sensitization after repeated NTG injection.

### PACAP and PAC1Rs were upregulated in TNC after repeated NTG administration

**Fig. 2:**
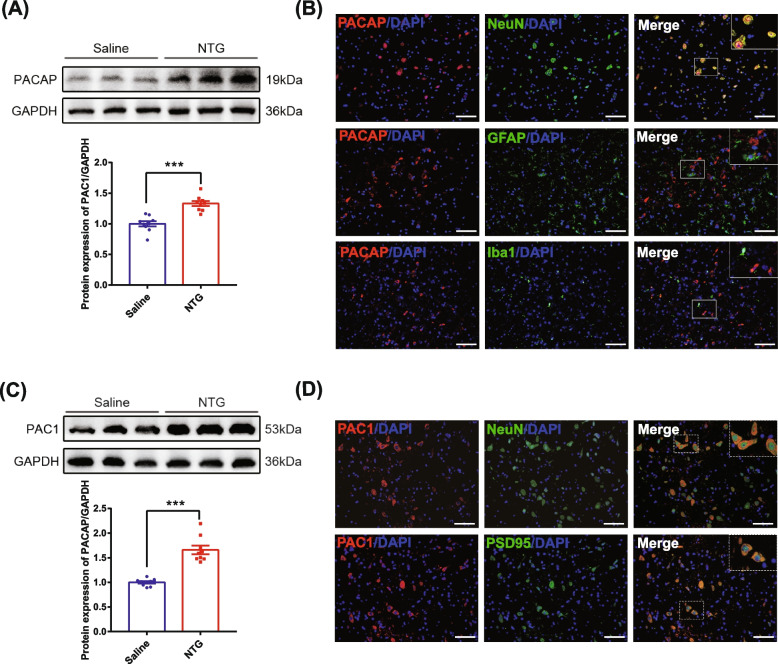
The protein expression and fluorescence localization of PACAP and PAC1R in different groups. **A**-**D**. Representative immunoblots and densitometric quantification of PACAP (**A**) and PAC1R (**C**) in the trigeminal nucleus caudalis (TNC). **B**. Representative microphotographs of co-immunofluorescence staining of PACAP (red) with neurons (NeuN, green), astrocytes (GFAP, green), and microglia (Iba1, green) in TNC after CM. The nuclei were stained with DAPI (blue). **D**. Representative microphotographs of co-immunofluorescence staining of PAC1R (red) with neurons (NeuN, green) and PSD95 (green) in TNC after CM. The nuclei were stained with DAPI (blue). Scale bar: 50 μm. Mean ± SEM, *n* = 3/group, two-tailed Student’s t-test, *p < 0.05, **p < 0.01, ***p < 0.001 compared with saline group. Abbreviations: NTG, nitroglycerin

To explore the effect of NTG administration on PACAP and PAC1R in TNC, we detected their protein expression levels by western blot in NTG and saline groups. The results showed that the expression of PACAP and PAC1Rs were significantly increased after repeated NTG injections (*p* < 0.001; Fig. 2A and C), which indicates that PACAP and PAC1R were activated in the TNC of CM rat. We further explored the cellular specificity of PACAP using double immunofluorescence staining. The PACAP was mainly expressed in NeuN-positive neurons (Fig. 2B), not co-localized with iba1-positive microglia and GFAP-positive astrocyte (Fig. 2B). In addition, double immunofluorescence staining showed that PAC1R was partially expressed in NeuN-positive neurons and colocalized with the PSD95 (Fig. 2D), a marker of the postsynaptic membrane (Fig. 2D). Based on these results, we conclude that the expression of PACAP and PAC1Rs was upregulated in the TNC after repeated NTG injection, and that PACAP/PAC1 signaling pathway may be engaged in the pathophysiological mechanisms of CM through regulating synapse-associated proteins.

### PAC1R inhibitor PACAP6-38 alleviated central sensitization and reduced neuronal activation in the TNC of CM rat model

**Fig. 3:**
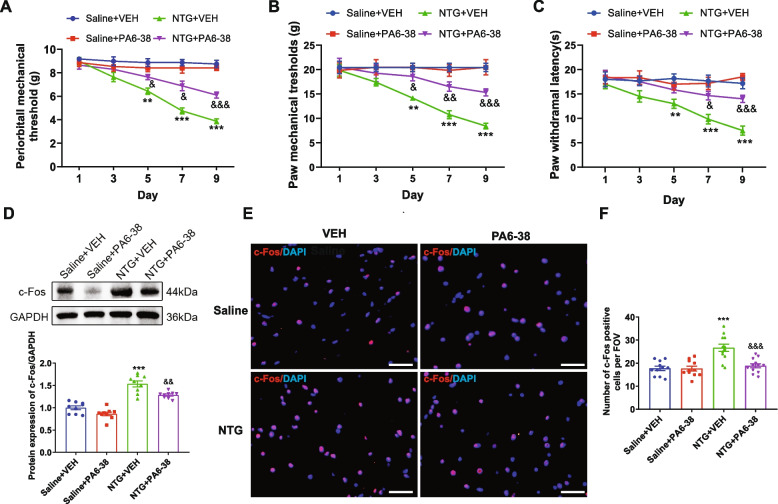
PAC1R inhibitor PACAP6-38 alleviated central sensitization and reduced neuronal activation in the TNC of CM rat model. **A**-**C**. PACAP6-38 treatment ameliorated the decreased mechanical (**A**-**B**) and thermal (**C**) thresholds after CM. Mean ± SEM, *n* = 6/group. Two-way ANOVA with the Bonferroni post hoc test, *p < 0.05, **p < 0.01, ***p < 0.001 compared with saline + vehicle group, ^&^*p* < 0.05, ^&&^*p* < 0.01, ^&&&^*p* < 0.001 compared with NTG + vehicle group. **D**. Representative western blot bands and densitometric quantification of c-Fos in different groups. **E**–**F**. Representative micrographs (**E**) and quantitative analysis (**F**) of c-Fos (red) staining in TNC. The nuclei were stained with DAPI (blue). Mean ± SEM, *n* = 3/group. One-way ANOVA, Dunnett; *p < 0.05, **p < 0.01, ***p < 0.001 compared with saline + vehicle group, ^&^*p* < 0.05, ^&&^*p* < 0.01, ^&&&^*p* < 0.001 compared with NTG + vehicle group. Abbreviations: PA6-38, pituitary adenylate cyclase-activating peptide 6–38; NTG, nitroglycerin; CM: chronic migraine

To further examine the role of PAC1R in the progression of central sensitization after CM, we administrated the PAC1R antagonist PACAP6-38 to rats 30 min before each NTG injection. Multiple pretreatments with PACAP6-38 markedly increased basal mechanical and thermal thresholds (Fig. 3A, B and C) compared with NTG + VEH group. Moreover, we examined c-Fos expression as a marker of neuronal activation after CM. In the NTG + PA6-38 group, protein expression of c-Fos was significantly decreased in TNC compared to the NTG + VEH group (*p* < 0.01; Fig. 3D). The results of immunofluorescence staining demonstrated that the number of c-Fos-positive cells in the TNC area of the NTG + PA6-38 group was significantly lower than that of the NTG + VEH group (*p* < 0.001; Fig. 3D). In conclusion, it was observed from the experimental results that inhibiting PAC1R with PACAP6-38 could alleviate the NTG-induced central sensitization and neuronal activation in TNC.

### PACAP6-38 administration decreased the overexpression of synaptic-associated proteins in CM rat model

**Fig. 4:**
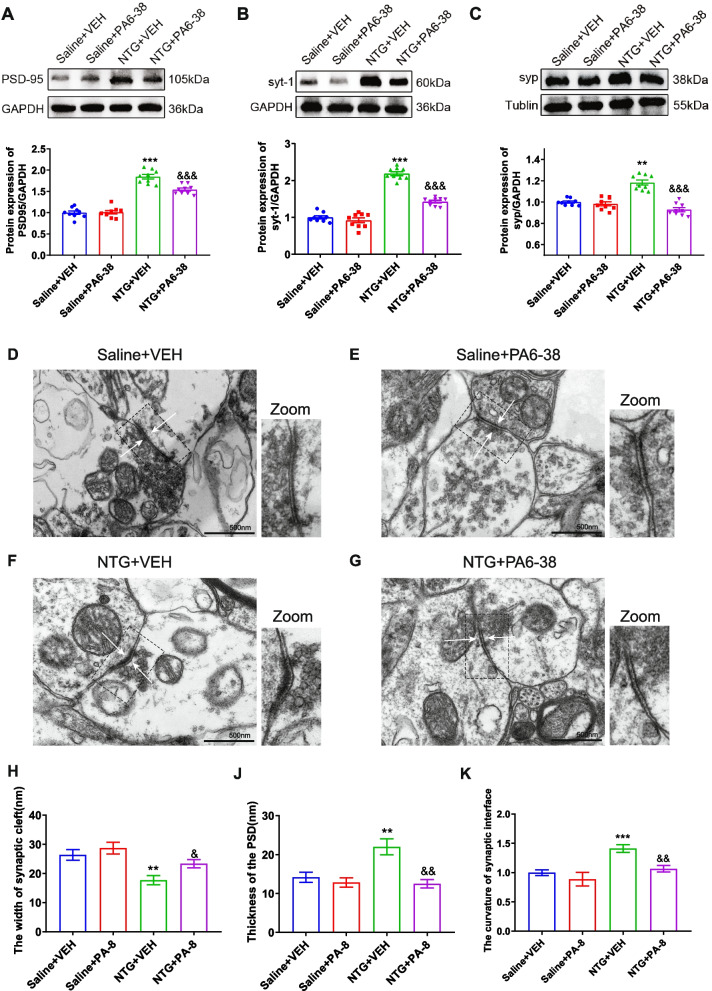
PACAP6-38 administration decreased the overexpression of synaptic-associated proteins and restored the aberrant synaptic ultrastructure in CM rat model. **A**-**C**. Representative bands and quantification of PSD-95, syt-1 and syp protein levels showed that these proteins were significantly increased in the NTG + vehicle group compared to the saline + vehicle group, while PACAP6-38 blocked these effects. **E**–**G**. Representative images of the synaptic ultrastructure in the four groups. **H**-**J**. The width of the synaptic cleft (**H**) was decreased, and the thickness of PSD (**I**) and the synaptic interface curvature (**J**) were significantly increased in the NTG + VEHgroup compared to the Saline + VEHgroup. In the NTG + PA6-38 group, these abnormal alterations were alleviated. Mean ± SEM. *n* = 3/group. One-way ANOVA with Dunnett’s post hoc test; Scale bars = 500 nm. *p < 0.05, **p < 0.01, ***p < 0.001 compared with saline + vehicle group, ^&^*p* < 0.05, ^&&^*p* < 0.01, ^&&&^*p* < 0.001 compared with NTG + vehicle group. Abbreviations: PA6-38, pituitary adenylate cyclase-activating peptide 6–38; NTG, nitroglycerin; CM: chronic migraine

Dysregulated synaptic plasticity can cause central sensitization, which is a key pathophysiological mechanism of CM [44]. Considering the colocalization of PAC1R with PSD95, we further investigated whether PACAP/PAC1R could regulate the expression of PSD95, syp and syt-1 in TNC. PSD95 is essential for synaptic maturation and dendritic spine development and stabilization [45]. The syp is a marker protein of synaptic vesicles and is altered by synaptic dysfunction [46]. Syt-1 is a multifunctional protein producing vesicle docking, excitation, and fusion [47]. The results of immunoblotting showed that the expression of PSD95, syt-1, and syp was greatly increased in the NTG + VEH group compared with that in the saline + VEH group, while repeated treatment with PACAP6-38 markedly reduced the expression of these three proteins in the NTG + PA6-38 group (*p* < 0.001; Fig. 4A-C).

### Inhibiting PAC1R with PACAP6-38 restored the aberrant synaptic ultrastructure and neuronal dendritic spines in CM rat model

Synaptic ultrastructure is vital for synaptic transmission and synaptic plasticity [48]. Therefore, we measured the width of the synaptic cleft, the thickness of the postsynaptic density (PSD) and the curvature of synaptic interface as morphological indexes by TEM in this experiment. We found that repeated NTG caused ultrastructural damage to synapses in the TNC. Ultrastructure in the NTG + VEH group was fuzzy and disorganized while in the Saline + VEH and NTG + PA6-38 group, synaptic ultra-structures were clear and relatively intact (Fig. 4D-G). The ultrastructural analysis of the synapses showed that NTG treatment increased the PSD thickness (Fig. 4H) and synaptic interface curvature (Fig. 4I), and decreased the width of the synaptic cleft (Fig. 4J) compared with those in the Saline + VEH group. Moreover, these abnormal alternations were significantly alleviated after repeated PACAP6-38 treatment (Fig. 4H-J).

**Fig. 5:**
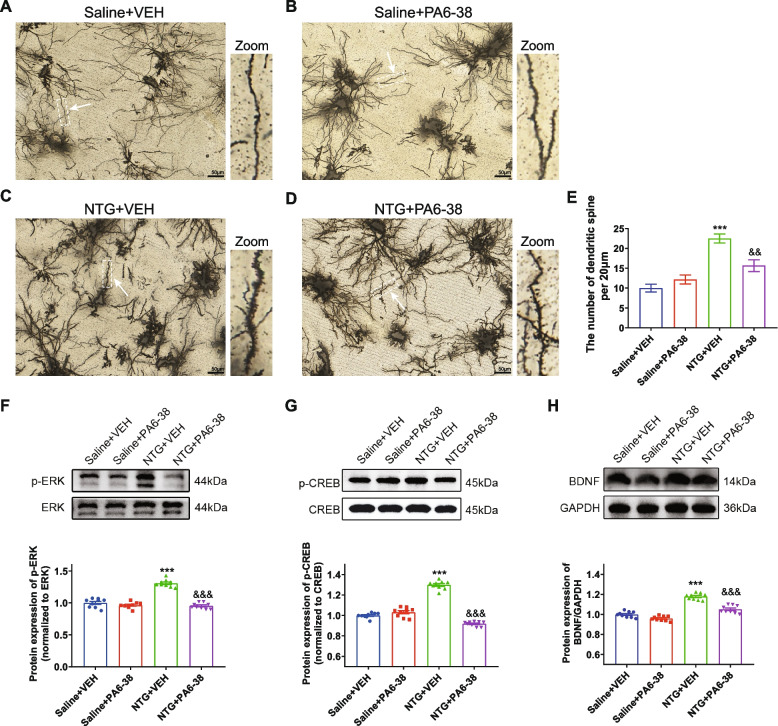
Inhibiting PAC1R with PACAP6-38 restored the aberrant neuronal dendritic spines via the ERK/CREB/BDNF signaling pathway in the CM rat model. **A**-**D**. Representative images of the dendritic spines in different groups. **E**. Golgi-Cox staining showed that the number of dendritic spines per 20 μm was significantly higher in the NTG + VEH group compared to the Saline + VEH group, and the effect was inhibited in the NTG + PA6-38 group. (F–H). Representative bands showed that the protein levels of p-ERK(**F**), p-CREB (**J**) and BDNF (**H**) were significantly elevated in the NTG + VEH group compared to the Saline + VEH group. After PACAP6-38 treatment, the expression levels of these proteins were significantly reduced. Mean ± SEM, *n* = 3/group. One-way ANOVA with Dunnett’s post hoc test; *p < 0.05, **p < 0.01, ***p < 0.001 compared with saline + vehicle group, ^&^*p* < 0.05, ^&&^*p* < 0.01, ^&&&^*p* < 0.001 compared with NTG + vehicle group. Abbreviations: PA6-38, pituitary adenylate cyclase-activating peptide 6–38; NTG, nitroglycerin; CM: chronic migraine

In addition, we examined the modification of dendritic spines in TNC, which is assumed as the main postsynaptic sites of excitatory input [49]. The Golgi staining results revealed that the number of dendritic branches in the NTG + VEH group was higher than that in the Saline + VEH group (*p* < 0.001, Fig. 5A, C and E). The density of dendritic spines was considerably reduced after several PACAP6-38 treatments (*p* < 0.01, Fig. 5E).

### Inhibiting PAC1R with PACAP6-38 regulated the ERK/CREB/BDNF signaling pathway in the CM rat model

Previous articles reported that the ERK/CREB/BDNF signaling pathway has a critical role in long-term synaptic plasticity in the hippocampus, particularly in diseases of cognitive impairment [50]. It is yet unknown, nevertheless, if the ERK/CREB/BDNF signaling is a possible route involved in the PACAP/PAC1R-mediated regulation of synaptic transmission in the TNC following CM. We next explore the effect of PAC1R inhibitor on the ERK/CREB/BDNF pathway through western blot. In the NTG + PA6-38 group, PACAP6-38 significantly blocked the increased expression of p-CREB/CREB (*p* < 0.001; Fig. 5F), p-ERK/ERK (*p* < 0.001; Fig. 5G) and BDNF (*p* < 0.001; Fig. 5H) in the TNC of CM rats, compared with the NTG + VEH group. The results lend credence to the hypothesis that PACAP6-38 may influence synaptic transmission in CM rats involved the ERK/CREB/BDNF signaling pathway.

## Discussion

The purpose of this study was to explore whether intracerebral injection of the antagonist PACAP6-38 improves central sensitization in TNC of CM rats. Many clinical studies indicated that intravenous PACAP could trigger a delayed migraine-like attack [12, 51], meaning the excitatory function of PACAP in pain transmission. The effects of PACAP are mediated through three different GPCRs: VPAC1, VPAC2 and PAC1R [52]. PACAP6-38 is a truncated version of PACAP, which is commonly used as a PAC1R antagonist but also blocks VPAC2R signaling and exhibits partial agonist activity [35, 53]. In our study, no neuronal activation and hypersensitivity was observed with PACAP6-38 alone (Fig. 3), suggesting that partial agonism may not be a complicating factor. Among three GPCRs, PAC1R binds PACAP with 1000 × higher affinity than VIP, while VPAC1 and VPAC2 have similar affinity for PACAP and VIP [54]. Because of its selectivity, PAC1R appears to be the most suitable target for migraine therapy. Akerman et al. [37] indicated intracerebroventricular injection of PACAP6-38 (3ug in 3ul), but not intravenous injection (150ug/kg), was able to cause significant inhibition of dural-evoked trigeminovascular neuronal responses. Recently PAC1 mAbs failed to pass a phase II clinical trial [55]. This result may be explained by the CNS effect of PACAP-induced migraine [37], as the anti-PACAP mAbs fail to cross the blood–brain barrier (BBB). Furthermore, a recent study showed that intraperitoneal administration of anti-PACAP mAb did not work in NTG model [56]. Therefore, in the present study, 3 μl of PACAP6-38 was microinjected locally into the TNC with a dose of 1 μg/ul to avoid it acting outside the brain.

**Fig. 6:**
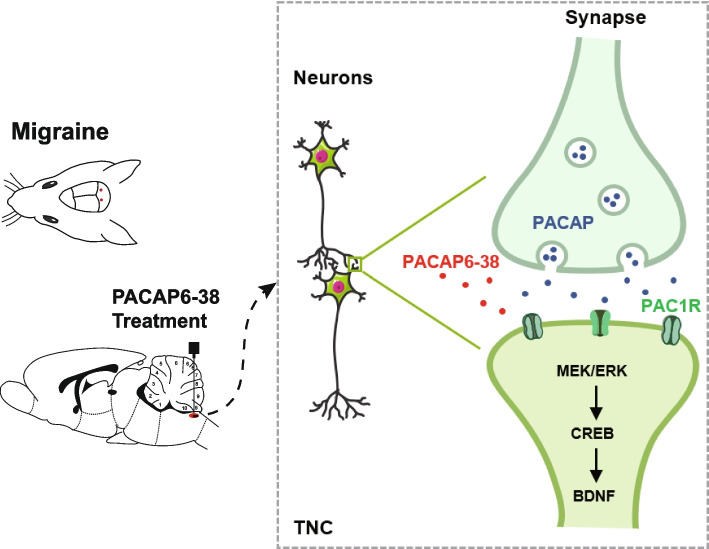
The graphical Abstract of this study. PACAP/PAC1R may contribute to NTG-induced central sensitization by regulating synaptic plasticity via ERK/CREB/BDNF signaling in CM model. PACAP modulates receptors on neuronal cells and enhances the activation of signaling pathways (ERK) and key transcription factors (CREB), which regulate the synthesis of synapse-associated proteins and adjust synaptic ultrastructure in the TNC. Abbreviations: PACAP, pituitary adenylate cyclase-activating peptide. NTG, nitroglycerin; CM: chronic migraine; TNC, trigeminal nucleus caudalis

Our results showed that the expression of PACAP and PAC1R was obviously elevated in TNC after NTG injection. PACAP was primarily co-expressed with neurons in TNC, and PAC1R was co-localized with the postsynaptic membrane in TNC. Treatment with PACAP6-38 could ameliorate NTG-induced dropping of baseline thresholds and alleviate central sensitization, with this process related to the down-regulation of synaptic plasticity-related proteins and restoration of the abnormal synaptic structural changes. Inhibition of PAC1R reduced the expression of BDNF, and phosphorylation of ERK and CREB in TNC after CM. Taken together, these results indicated PACAP/PAC1R may contribute to central sensitization by regulating synaptic plasticity via ERK/CREB/BDNF signaling in NTG-induced CM model (Fig. 6).

According to the previous study [21], we established a validated CM model in rats by repeated intraperitoneal injections of NTG. Repeated NTG injections can produce a significant time and dose-dependent chronic basal mechanical and thermal hyperalgesia, which is the hallmark of successful CM rat model construction [57, 58]. Given that the expression of PACAP differs significantly in the estrous cycle of female rats, male SD rats were selected for this study [59, 60]. Furthermore, the up-regulation of c-Fos in TNC after CM can be used as a sign for central sensitization [43]. NTG is regarded as an NO donor that mediates its mechanism of triggering migraine-like attacks. Although the NTG model of migraine is reliable and widely used, it nonspecifically mediates the endogenous release of multiple neuropeptides, including PACAP and CGRP. There are limitations in exploring the mechanisms of PACAP-mediated activation and sensitization of trigeminovascular neurons in the NTG model due to the overlap in NTG-mediated neurotransmitter mechanisms.

Central sensitization is hallmarked by enhanced excitability of neurons in nociceptive pathways and enhanced synaptic transmission [61, 62]. Synapses are places where neurons connect and communicate with each other. As a result of different physical and pathological conditions, synapses can change in their number, structure, and degree of activity, which is known as synaptic plasticity [63]. Several in vivo and in vitro studies have shown that exogenous PACAP could increase excitatory synaptic transmission in the hippocampal, which can be inhibited by PACAP6-38 [26, 27, 64]. Therefore, we speculated that PACAP may participate in the NTG-induced central sensitization by altering neural synaptic plasticity in TNC of CM rats. Our results of double immunofluorescence staining showed that PAC1R co-localized with PSD95, suggesting that PACAP can exert postsynaptic effects. PSD95 is a key postsynaptic scaffolding protein in excitatory neurons, which plays an important role in synaptic efficiency transmission by forming signal complexes with N-methyl-D-aspartate (NMDA) receptors or potassium channels to maintain and modulate synaptic plasticity [65]. In addition, our results showed that PACAP6-38 inhibited the expression of several synaptic associated-proteins, including PSD95, Syp, and Syt-1, in TNC after CM. We also examined the synaptic ultrastructure and the density of neuronal dendritic spines in TNC neurons of CM rats, which are strongly correlated with synaptic transmission efficiency. The results showed that PACAP6-38 treatment could improve the abnormal synaptic ultrastructure and dendritic branches.

To identify underlying mechanisms of neuronal activation in the TNC, we further explored the downstream pathways. A few studies provided evidence that activation of PAC1R can stimulate different pathways to enhance NMDA receptor function in hippocampal neurons [64]. The ERK signaling cascades play an essential role in NMDAR-dependent neuronal plasticity and survival [66, 67]. Phosphorylated CREB protein promotes the late phase of LTP by enhancing synaptic capture [68]. BDNF is known to be the most enriched neurotrophic factor in brain and regulated by p-CREB [32, 33]. Therefore, the synthesis of BDNF rises when synaptic activity increases. Consistent with previous results, our results showed that p-ERK, p-CREB, and BDNF expression levels significantly increased after NTG injection, and PACAP6-38 reversed the effects. These results indicated that PACAP6-38 is capable of improving synaptic transmission and neuronal activation involved ERK/CREB/BDNF in CM rats.

## Limitations of the study

There are several limitations in our study. Migraine is more frequent in females than males, and sexual dimorphism in migraine pathophysiology has been reported. However, the expression level of PACAP is affected by estrogen, and to avoid the fluctuation of PACAP across the estrous cycle in female rats, we used only male rats in this study. Furthermore, additional experiments are needed to explore the role of PACAP/PAC1R in central sensitization caused by PACAP or a selective PAC1R agonist. Also, we only focused on ERK/CREB/BDNF signaling pathway, but other signaling required more exploration. Then, the involvement of GABAergic interneurons in the long-term effects of PACAP6-38 treatment is still to be investigated. Finally, we only examined the impact of morphological alternations on synaptic transmission, and further electrophysiological experiments are necessary to investigate the influence on synaptic function.

## Conclusion

In summary, our study indicated that repeated NTG administration in rat upregulated the expression of both PACAP and PAC1R in TNC. Furthermore, PACAP6-38, a specific PAC1R antagonist, impaired the enhancement of neuronal activity and alleviated the chronic cephalic allodynia by modulating NTG-induced synaptic plasticity via ERK/CREB/BDNF pathway. Our findings may shed new light on inhibiting PACAP/PAC1R as potential therapeutic targets for migraine.

## Data Availability

The data that support the findings of this study are available from the corresponding author upon request.
